# Supplementary material for: PERK-Mediated eIF2α Phosphorylation Contributes to The Protection of Dopaminergic Neurons from Chronic Heat Stress in Drosophila
Source: Int J Mol Sci. 2020 Jan 28;21(3):845. doi: 10.3390/ijms21030845 (PMC7037073; doi:10.3390/ijms21030845)
Supplement: Supplementary file 1 [file ijms-21-00845-s001.pdf]

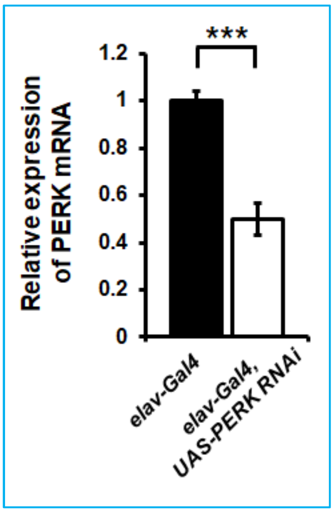

Figure S1. Knockdown of PERK by *elav-Gal4* in *Drosophila* neurons. Quantitative RT-PCR results confirmed the significantly decreased expression level of *PERK* mRNA. qRT-PCR was performed using total RNA extracted from heads. Error bars represent mean ± standard deviation of three independent experiments. The experimental significance was determined using a one-way ANOVA (***$p < 0.01$).
